# Transcriptome and Metabolome Dissection of Multilayered Pydiflumetofen Resistance Mechanisms in *Fusarium graminearum*

**DOI:** 10.3390/ijms27156685

**Published:** 2026-07-27

**Authors:** Yun Wang, Dongmei Liu, Haiyan Yin, Cheng Cao, Yingni Cao, Dan Feng, Guanghua Zhao, Junyan Wang, Hongxia Shang, Hongqi Wang, Jihong Liu

**Affiliations:** Institute of Quality and Safety for Agro-Products, Henan Academy of Agricultural Sciences, Zhengzhou 450002, China; wangyun1906@163.com (Y.W.); dongmeiliu80@163.com (D.L.); yinhaiyan2005@126.com (H.Y.); lllaohucao@163.com (C.C.); caoyingni0501@163.com (Y.C.); freefd89@sina.com (D.F.); zhaohua79@sina.com (G.Z.); wjyan1735@163.com (J.W.); ylshhx@163.com (H.S.)

**Keywords:** *Fusarium graminearum*, succinate dehydrogenase subunit C, transcriptomics, metabolomics, pydiflumetofen

## Abstract

Wheat Fusarium head blight (FHB) is a globally prevalent and destructive fungal disease predominantly caused by *Fusarium graminearum*. Pydiflumetofen, a novel succinate dehydrogenase inhibitor (SDHI) fungicide, exhibits strong inhibitory activity against *F. graminearum*; however, the molecular regulatory mechanisms underlying the field-developed resistance in this pathogen remain poorly defined. In the present study, a field-evolved resistant isolate W24-039 and a sensitive isolate W24-016 were subjected to multi-omics analysis. The sequencing results identified compound mutations C89S/A93V in SdhC2, and A21T/S30F in SdhD of the resistant strain, which confer stable fungicide resistance without any detectable fitness costs. Physiological tests revealed that these target mutations sustain the homeostasis of succinate dehydrogenase (SDH) activity and intracellular ATP production. Following pydiflumetofen treatment, the sensitive isolate displayed remarkable declines in SDH activity, intracellular ATP content and deoxynivalenol (DON) biosynthesis, accompanied by markedly elevated cell membrane permeability. Transcriptomic sequencing uncovered 2221 differentially expressed genes (DEGs) in the sensitive strain under fungicide stress, and 2566 DEGs in the resistant isolate under the same conditions. The genes associated with detoxification and drug efflux, including cytochrome P450, glutathione S-transferase (GST), ABC and MFS transporters, were significantly upregulated in the resistant isolate. Metabolomic analysis indicated that differential metabolites were mainly enriched in the tricarboxylic acid (TCA) cycle, amino acid metabolism and membrane lipid biosynthesis pathways. The resistant strain maintained intact TCA cycle operation and accumulated high levels of pivotal metabolites such as phosphatidylcholine, unsaturated fatty acids and reduced glutathione. Integrated multi-omics analysis verified that the ABC transporter and glutathione metabolism pathways serve as core regulatory modules governing fungicide resistance. Collectively, *F. graminearum* develops resistance via the synergistic effects of SDH compound mutations, enhanced detoxification and efflux, and global metabolic remodeling, demonstrating that target-site mutation alone is not the sole driver of resistance, which is instead controlled by an intricate regulatory network involving multiple coordinated pathways. This study systematically characterizes the resistance regulatory network of *F. graminearum* against pydiflumetofen, and provides theoretical guidance for the rational application and sustainable field resistance management of this fungicide.

## 1. Introduction

*Fusarium graminearum* is a devastating fungal pathogen responsible for Fusarium head blight (FHB) in wheat and other small grain cereals worldwide. Beyond causing severe yield losses, this pathogen contaminates grains with mycotoxins, predominantly deoxynivalenol (DON). DON not only poses a significant threat to food and feed safety, but also acts as a crucial virulence factor, actively facilitating fungal colonization and spread within host tissues [1,2]. Given the paucity of highly resistant wheat cultivars, chemical control remains the most effective and widely implemented strategy for FHB management. Within this context, succinate dehydrogenase inhibitors (SDHIs) have emerged as a critical class of fungicides due to their unique mode of action and broad-spectrum antifungal activity. Among them, the novel SDHI pydiflumetofen has been extensively deployed in field applications owing to its potent inhibitory efficacy against the target enzyme succinate dehydrogenase (SDH) [3,4].

SDHIs exert their fungicidal activity by blocking ubiquinone binding at complex II, thereby disrupting electron transport and energy homeostasis [5,6]. However, continuous selection pressure has inevitably driven the emergence of pydiflumetofen-resistant *F. graminearum* field isolates. Current evidence firmly establishes that structural alterations—primarily non-synonymous point mutations within SDH subunits (*SdhB*, *SdhC*, and *SdhD*)—represent the core mechanism conferring resistance by significantly impairing fungicide–target affinity [7,8]. While such mutations effectively elevate resistance levels, laboratory and field observations indicate that these primary target-site modifications frequently incur fitness penalties, compromising vegetative growth and pathogenicity [9,10]. This observation suggests a fundamental physiological trade-off: the very mutations that enable survival under fungicide stress can simultaneously disrupt intrinsic metabolic homeostasis.

Despite this clear understanding of the genetic basis, the prevailing “linear paradigm” of SDHI resistance—attributing insensitivity solely to impaired local drug–target binding—fails to capture the pathogen’s holistic adaptive strategy. Evolving resistance is not merely a localized structural event, but a complex, systemic adaptive trait. Sustaining viability under acute xenobiotic stress requires profound, genome-wide transcriptional reprogramming and robust metabolic rewiring that extends far beyond the target gene itself [11]. The frequent observation of physiological trade-offs in resistant strains strongly implies that primary target mutations initiate cascading perturbations in central carbon metabolism, redox balance, and membrane homeostasis [9,12]. Nevertheless, how pathogens systematically remodel their global metabolic networks to compensate for these pleiotropic effects and how this remodeling mechanistically integrates with the resistance phenotype remain largely uncharacterized.

The rapid advancement of omics technologies has provided powerful tools for globally interrogating organismal stress responses and adaptive mechanisms. The integrated analysis of transcriptomic and metabolomic data enables the construction of a coherent framework linking “gene transcriptional regulation” to “metabolic phenotypic outputs”, thereby facilitating the multi-dimensional dissection of complex biological processes [13]. In the field of plant–pathogen–fungicide interactions, multi-omics integration strategies have successfully delineated xenobiotic detoxification networks and energy compensatory pathways underlying resistance to other fungicide classes, such as quinone outside inhibitors (QoIs) and demethylation inhibitors (DMIs) [14,15]. For *F. graminearum*, comparative transcriptomic analyses have revealed that genes involved in carbohydrate metabolism, amino acid metabolism and lipid metabolism exhibit significant differential expression under various fungicide stresses, confirming the central role of core carbon metabolic pathways in mediating fungicide responses [16]. However, comprehensive investigations interrogating the coordinate multi-omics architecture of SDHI-resistant *F. graminearum* isolates, particularly those that systematically compare the divergent adaptive strategies of resistant versus sensitive strains under pydiflumetofen challenge, remain remarkably scarce.

Our previous population surveillance study, spanning 2021–2024, collected 345 *F. graminearum* isolates across Henan Province, systematically profiling baseline sensitivity, resistance frequency, and cross-resistance patterns against pydiflumetofen [17]. However, the multi-dimensional adaptive strategies employed by naturally evolved resistant versus sensitive field isolates under pydiflumetofen challenge remain unknown. To decipher this multi-dimensional resistance landscape, we conducted a comparative multi-omics investigation using a field-evolved pydiflumetofen-resistant isolate (W24-039) and its sensitive isolate (W24-016). By integrating transcriptomic and metabolomic analyses, we systematically dissected the synergistic resistance architecture: (i) target-site mutations in SdhC2 and SdhD sustaining energy homeostasis; and (ii) a coordinated cellular defense network comprising enhanced xenobiotic detoxification, active drug efflux and global metabolic reprogramming. This framework provides a molecular blueprint of SDHI resistance for guiding rational fungicide deployment.

## 2. Results

### 2.1. Sensitivity of F. graminearum to Pydiflumetofen and Target Mutations

**Figure 1:**
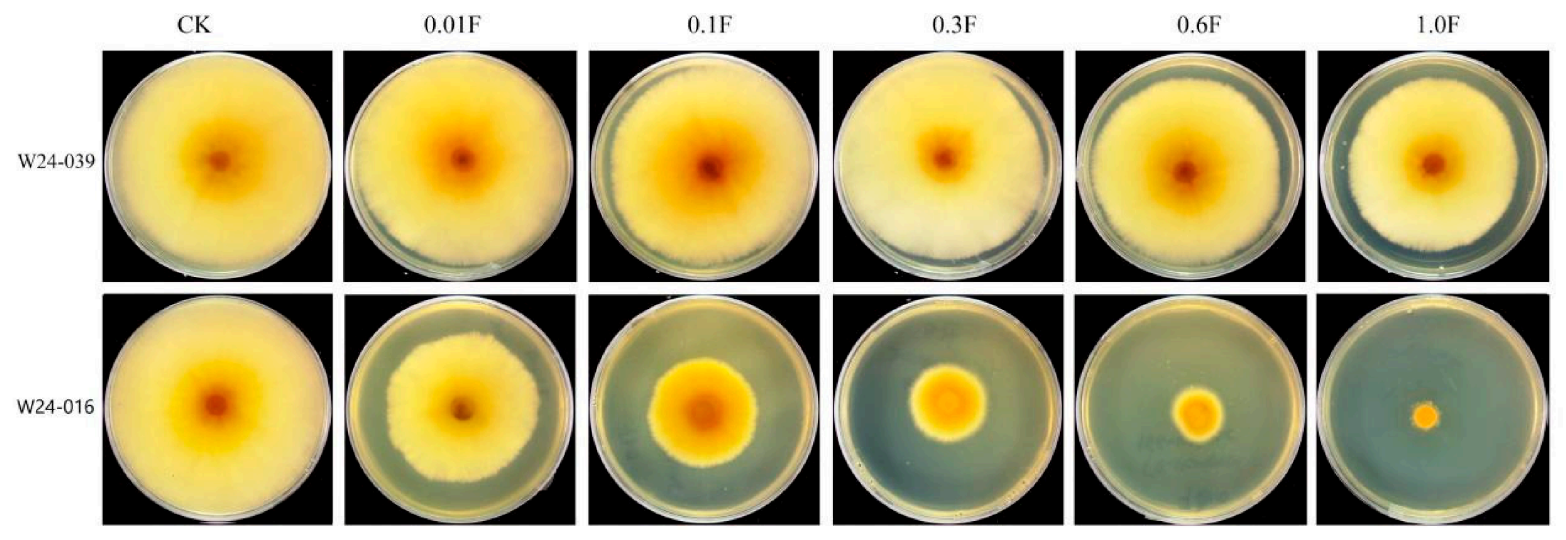
Colony morphology of resistant strain W24-039 and sensitive strain W24-016 cultured on pydiflumetofen-amended PDA plates. Note: CK indicates the fungicide-free control treatment; 0.01F, 0.1F, 0.3F, 0.6F and 1.0F correspond to media containing 0.01, 0.1, 0.3, 0.6 and 1.0 μg/mL pydiflumetofen, respectively.

In vitro sensitivity assays revealed striking phenotypic divergence between the two *F. graminearum* isolates (Table 1). The resistant isolate (W24-039) exhibited an EC_50_ value of 2.460 μg/mL, corresponding to a resistance factor (RF) of 31.94 relative to the sensitive isolate (W24-016; EC_50_ = 0.077 μg/mL). The targeted sequencing of five genes encoding all subunits of the SDH complex (FgSdhA, FgSdhB, FgSdhC1, FgSdhC2 and FgSdhD) identified unique compound mutations exclusively in the resistant isolate W24-039, which carried dual amino acid substitutions in FgSdhC2 (C89S/A93V) and FgSdhD (A21T/S30F), while no amino acid variants were detected in FgSdhA, FgSdhB and FgSdhC1. Mycelial growth assays on pydiflumetofen-amended PDA plates were performed at 25 °C in darkness for 7 days; strain W24-016 displayed severe growth inhibition, while strain W24-039 maintained robust growth, consistent with its resistant phenotype (Figure 1).

### 2.2. Fitness Assessment

Fitness parameters, including mycelial growth rate, sporulation, conidial germination and pathogenicity on wheat spikes, were compared between the resistant (W24-039) and sensitive (W24-016) isolates under fungicide-free conditions. No statistically significant differences were detected for any of these traits (Table 2). These results suggest that the SdhC2/SdhD compound mutations confer pydiflumetofen resistance without major fitness penalties under the assayed conditions, corroborating our earlier findings [17].

### 2.3. Physiological Responses to Pydiflumetofen Stress

**Figure 2:**
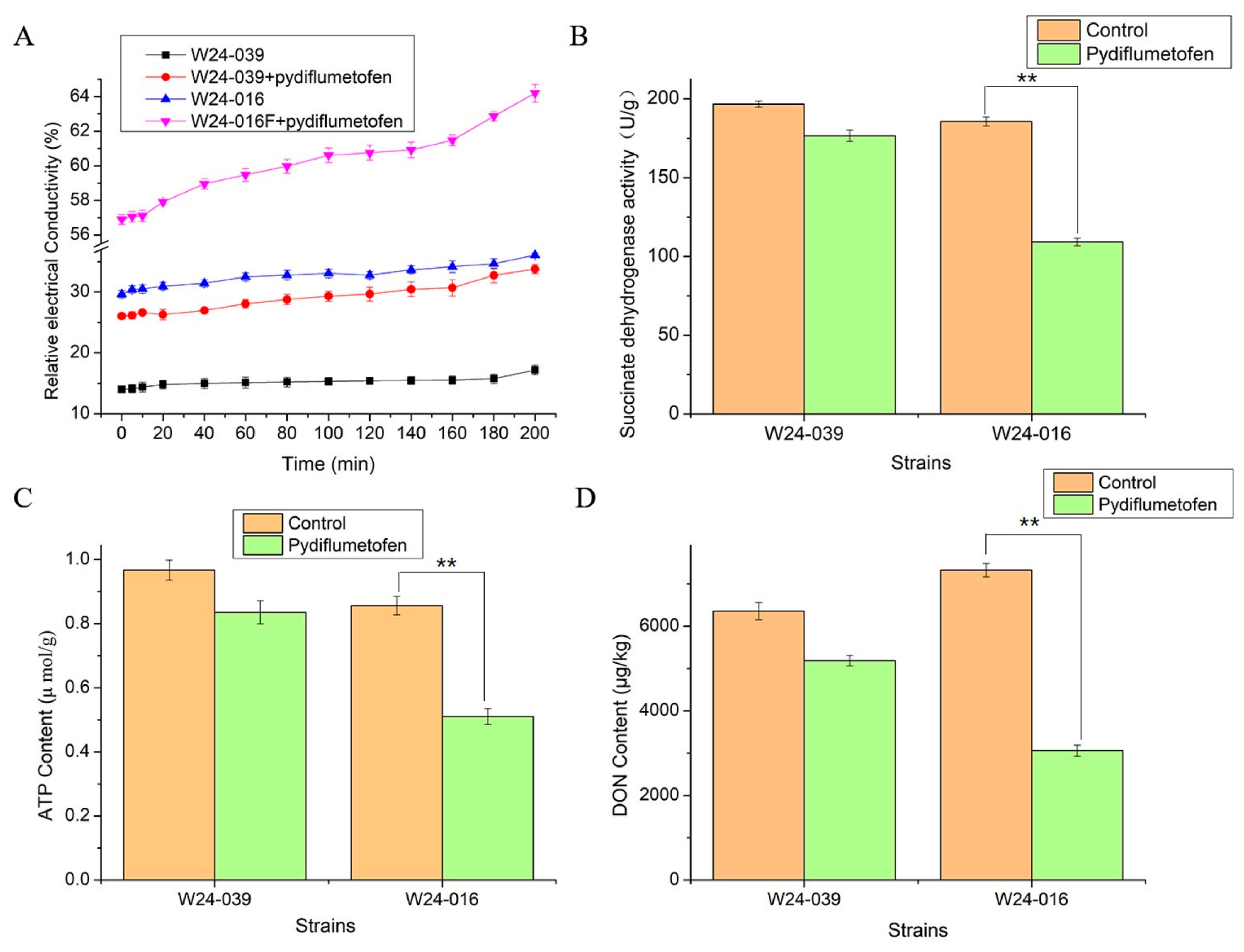
Physiological responses of pydiflumetofen-sensitive (W24-016) and resistant (W24-039) *F. graminearum* isolates under fungicide stress. (**A**) Dynamic changes in relative electrical conductivity; (**B**) succinate dehydrogenase (SDH) activity; (**C**) intracellular ATP content; (**D**) DON mycotoxin production. Data are presented as the mean ± standard deviation. ** indicates significant difference at *p* < 0.01 level between two groups.

To assess the physiological disruptions caused by pydiflumetofen stress, cell membrane integrity, energy metabolism and mycotoxin biosynthesis were evaluated (Figure 2). Under non-treated conditions, both isolates maintained comparable baseline levels across all indices. However, upon pydiflumetofen exposure, the sensitive isolate exhibited a significant temporal escalation in relative electrical conductivity, indicating severe cell membrane impairment and electrolyte leakage. Conversely, the resistant isolate sustained membrane integrity. Crucially, pydiflumetofen treatment drastically suppressed SDH enzymatic activity, intracellular ATP accumulation and deoxynivalenol (DON) production in the sensitive isolate. In stark contrast, the resistant isolate harboring the SdhC2/SdhD compound mutations demonstrated remarkable physiological resilience, maintaining these indices at levels comparable to the untreated control.

### 2.4. Transcriptomic Analysis

#### 2.4.1. RNA-Seq Data Quality and Mapping

Twelve cDNA libraries were constructed and sequenced, generating a total of 98.58 Gb of clean data, with an average of 8.21 Gb per sample. The Q30 values exceeded 96.7% for all samples, and more than 84.7% of the clean reads were uniquely mapped to the *F. graminearum* reference genome (PH-1, ASM24013v3) (Tables S1 and S2), confirming the high quality and reliability of the transcriptomic data for subsequent analysis.

#### 2.4.2. Overview of Differentially Expressed Genes (DEGs)

**Figure 3:**
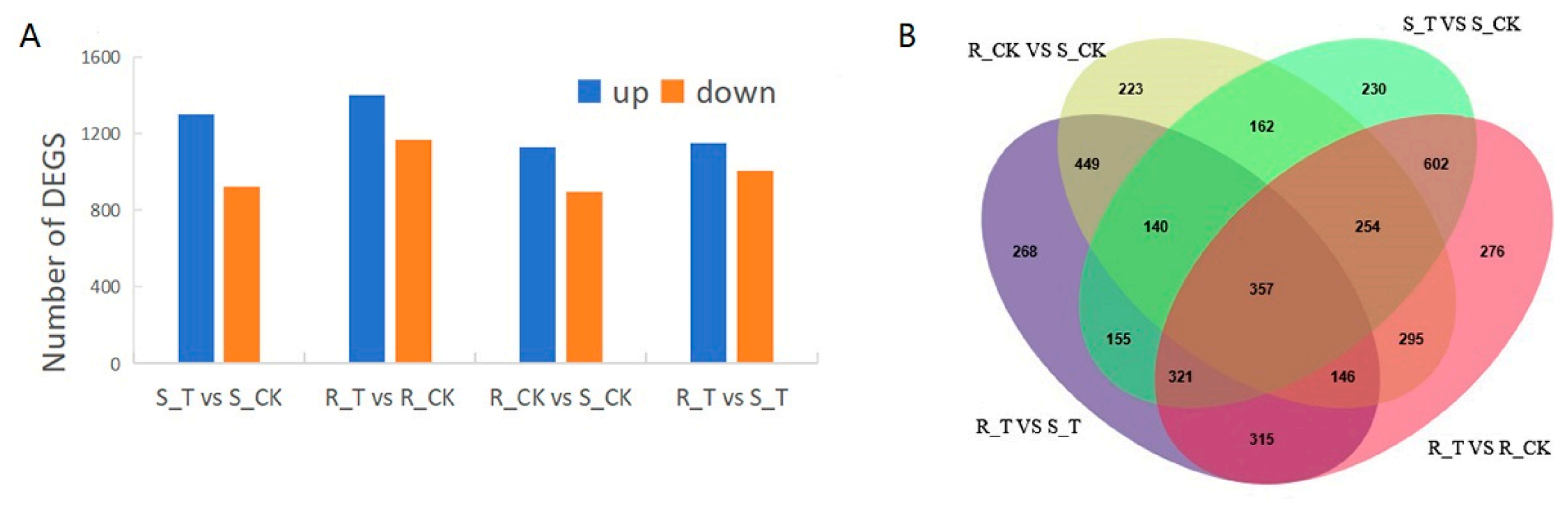
Summary of differentially expressed genes (DEGs) in four comparison groups. (**A**) Number of upregulated and downregulated DEGs in S_T vs. S_CK, R_T vs. R_CK, R_CK vs. S_CK and R_T vs. S_T; (**B**) Venn diagram analysis of shared and specific DEGs among four pairwise comparisons. S: sensitive; R: resistant; CK: untreated; T: treated.

To elucidate the transcriptional reprogramming underlying pydiflumetofen resistance, we performed pairwise comparisons among the four experimental groups (S_CK, S_T, R_CK, R_T). As shown in Figure 3A, fungicide treatment induced a more extensive transcriptional response in the resistant strain (2566 DEGs; 1399 up, 1167 down) than in the sensitive strain (2221 DEGs; 1297 up, 924 down). The comparison of the untreated controls (R_CK vs. S_CK) revealed 2026 DEGs (1130 up, 896 down), indicating that resistance-associated mutations profoundly altered the basal transcriptome. The direct comparison of the treated strains (R_T vs. S_T) identified 2151 DEGs (1147 up, 1004 down). Venn diagram analysis showed that 357 DEGs were commonly regulated across all four comparisons (Figure 3B), suggesting that these represent core resistance-associated genes of which the functional annotation is presented below. Full DEG information is provided in Supplementary Table S3.

#### 2.4.3. Functional Enrichment Analysis of DEGs

Gene Ontology (GO) functional enrichment analysis revealed (Figure S1) that all four comparison groups (S_T vs. S_CK, R_T vs. R_CK, R_T vs. S_T, and R_CK vs. S_CK) showed significant enrichment in pathways associated with transmembrane transporter activity and oxidoreductase activity within the category of molecular function. The R_CK vs. S_CK group was specifically enriched for pathways in the biological process, including rRNA processing, ribosome biogenesis and DNA-templated transcription. In addition to the common pathways, the R_T vs. S_T group was enriched for the ribosome in the cellular component, FMN binding and the structural constituent of ribosome in molecular function, as well as translation, proteolysis and response to oxidative stress in the biological process. The R_T vs. R_CK group exhibited the most abundant set of uniquely enriched pathways, encompassing flavin adenine dinucleotide binding and zinc ion binding in molecular function, GPI anchor biosynthetic process and the regulation of DNA-templated transcription in the biological process, and the extracellular region in the cellular component. The S_T vs. S_CK group was specifically enriched for pathways including carbon–sulfur lyase activity, quinone binding and O-methyltransferase activity in molecular function.

**Figure 4:**
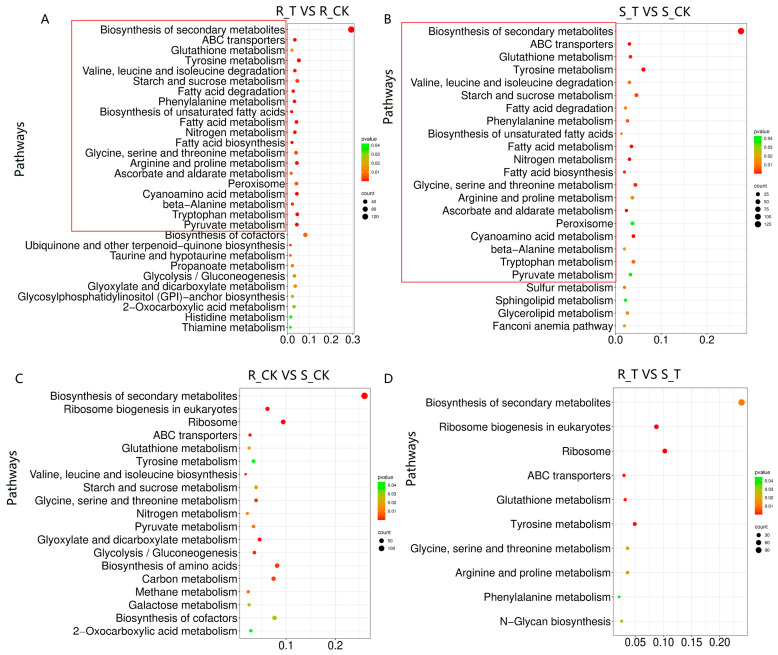
KEGG enrichment analysis of DEGs in (**A**) R_T vs. R_CK, (**B**) S_T vs. S_CK, (**C**) R_CK vs. S_CK and (**D**) R_T vs. S_T. The Padj value is displayed on the vertical axis, while the enrichment rate is on the horizontal axis. The size of each bubble indicates the number of enriched transcripts, which represents a KEGG pathway. Only pathways with Padj < 0.05 are displayed.

KEGG signaling pathway enrichment analysis revealed numerous significantly enriched pathways (*p* < 0.05) shared by both S_T vs. S_CK and R_T vs. R_CK comparisons (Figure 4), such as the ABC transporter pathway, glutathione metabolism pathway, tyrosine metabolism, starch and sucrose metabolism pathway, fatty acid degradation pathway, and valine, leucine, and isoleucine degradation pathway, among others. These pathways act as core shared defense mechanisms enabling strains to cope with fungicide stress. Meanwhile, the ribosome pathway and ribosome biogenesis pathway were significantly enriched in the R_CK vs. S_CK, R_T vs. S_T, and S_T vs. S_CK comparisons, with significant upregulation observed in the first two comparisons and obvious downregulation in the latter comparison.

Further analysis of group-specific enriched pathways revealed that the R_T vs. R_CK comparison exhibited a unique enrichment of pathways including glycosylphosphatidylinositol (GPI)-anchor biosynthesis, histidine metabolism, propanoate metabolism, taurine and hypotaurine metabolism, thiamine metabolism, ubiquitone and other terpenoid–quinone biosynthesis, ascorbate and aldarate metabolism, glyoxylate and dicarboxylate metabolism, glycolysis/gluconeogenesis, 2-oxocarboxylic acid metabolism, and valine, leucine and isoleucine degradation. GPI-anchor enrichment suggests enhanced cell wall integrity; concurrent glycolysis/gluconeogenesis and glyoxylate/dicarboxylate enrichment suggest a TCA bypass sustaining ATP production; and taurine, thiamine, ubiquinone and ascorbate pathways collectively imply boosted antioxidant defense and redox homeostasis. These enrichments depict a coordinated reprogramming sustaining energy production, redox balance, and cellular integrity in the resistant strain under fungicide stress.

The S_T vs. S_CK comparison showed unique enrichment of the Fanconi anemia pathway, sphingolipid metabolism pathway, sulfur metabolism pathway, and glycerolipid metabolism pathway. Among these, the specific activation of the Fanconi anemia pathway directly confirmed that the fungicide caused severe genomic DNA damage in the sensitive strain. Meanwhile, the downregulation of sphingolipid metabolism may disrupt the integrity of membrane lipid microdomains, further exacerbating cell membrane damage and susceptibility. The R_CK vs. S_CK comparison uniquely enriched pathways including ribosome biogenesis, ribosome, oxidative phosphorylation, methane metabolism, galactose metabolism, valine, leucine and isoleucine biosynthesis, cofactor biosynthesis and 2-oxocarboxylic acid metabolism. This indicates that even under baseline conditions without fungicide stress, the resistant isolate exhibited constitutively higher transcript levels for oxidative phosphorylation genes, pointing to a higher baseline capacity for energy metabolism and protein synthesis than the sensitive isolate. The R_T vs. S_T comparison uniquely enriched the N-glycan biosynthesis pathway, reflecting enhanced ER protein glycosylation capacity in the resistant strain, which may facilitate the proper processing of the membrane transporters involved in detoxification and efflux.

#### 2.4.4. Screening and Expression Analysis of Genes Related to Metabolic Resistance

We next examined the expression of genes encoding the SDH complex, detoxification enzymes, drug efflux pumps and trichothecene biosynthetic enzymes.

**Figure 5:**
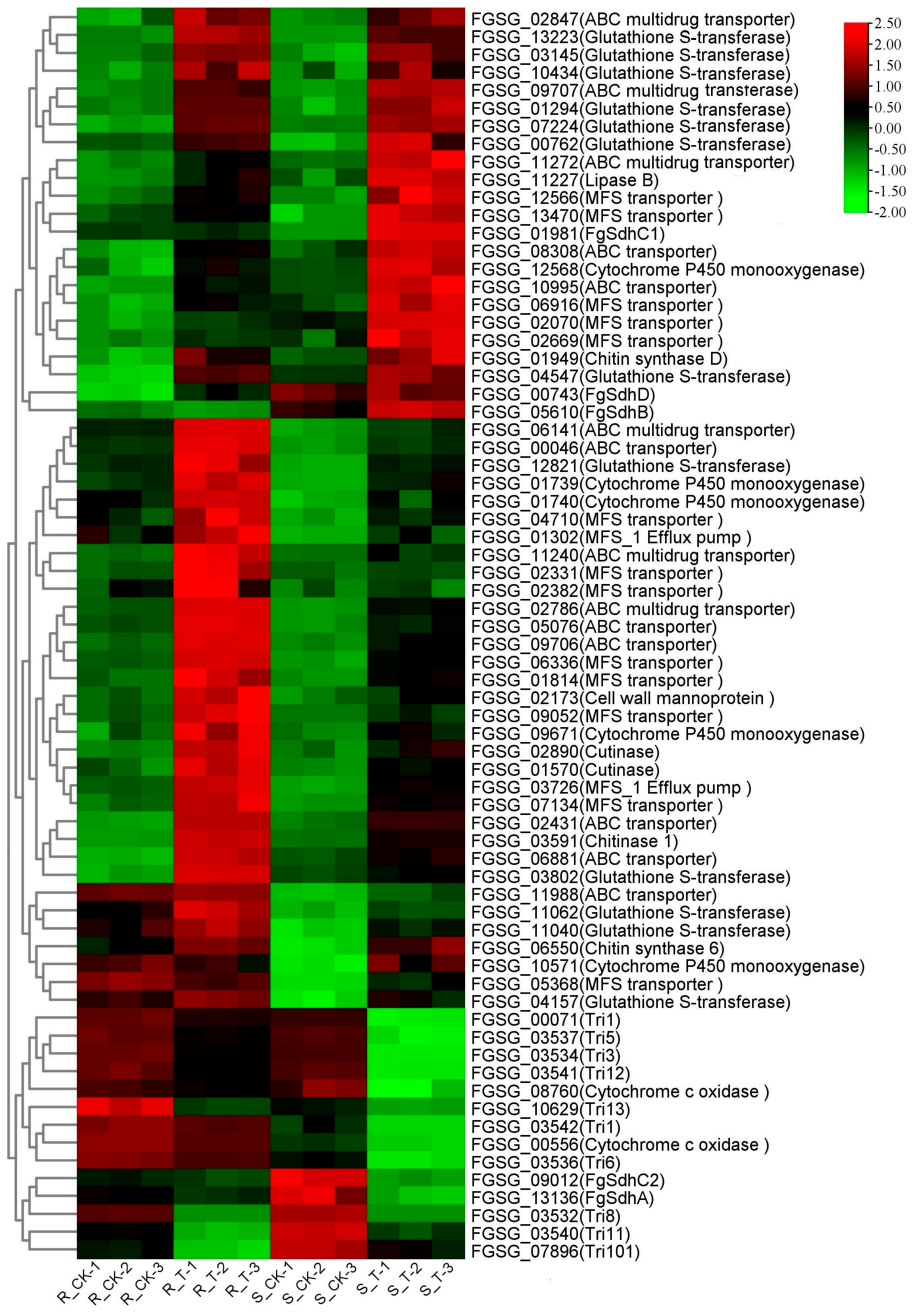
Heatmap of the DEGs implicated in resistance metabolic between R_T vs. R_CK and S_T vs. S_CK. Green indicates low expression levels and red indicates high expression levels. Colored blocks along the *y*-axis of the heatmap represent the family to which each gene belongs.

SDH complex genes. In the sensitive strain, pydiflumetofen treatment significantly upregulated the *FgSdhC1* subunit gene (FGSG_01981) (Figure 5), likely as a compensatory response to maintain respiratory function. No significant changes were observed for any SDH subunit genes in the resistant strain, consistent with the preservation of SDH activity conferred by the target-site mutations.

Detoxification and efflux genes. Cytochrome P450, glutathione S-transferase (GST), ABC transporters and major facilitator superfamily (MFS) transporters are essential for stress tolerance. ABC and MFS transporters are two major transmembrane transporter superfamilies, which function together with P450 and GST in defense against chemical, oxidative and other environmental stresses. In this study, 14 ABC genes and 15 MFS genes were significantly upregulated in *F. graminearum* upon pydiflumetofen treatment. Among them, six ABC transporters (*FGSG_02847*, *FGSG_09707*, *FGSG_06141*, *FGSG_11272, FGSG_11240*, *FGSG_02786*) and two MFS transporters (*FGSG_03726*, *FGSG_01302*) were annotated as multidrug efflux pumps, indicating that *F. graminearum* may reduce fungicide accumulation and toxicity by enhancing drug efflux. Detoxification genes *FGSG_13223* (GST) and *FGSG_01739* (cytochrome P450) were also markedly induced. Moreover, the expression levels of these efflux-related genes were higher in resistant strains than in sensitive strains. These results suggest that the synergistic function of efflux pumps and detoxification enzymes contributes to pydiflumetofen tolerance in *F. graminearum*.

*Tri* gene expression. In the sensitive strain, pydiflumetofen treatment significantly downregulated the key regulatory gene FgTri5 and the structural gene FgTri6 (log_2_FC = −2.8 and −2.1, respectively), consistent with a marked reduction in DON production (Figure 2D). In contrast, the resistant strain exhibited only minor, statistically non-significant downregulation of FgTri5 and FgTri6 (log_2_FC = −0.6 and −0.4, respectively), which correlated well with its stable DON levels under fungicide stress. Other Tri genes (FgTri13, FgTri3, FgTri12) showed similar trends. These results indicate that the resistance-conferring mutations in SDH subunits prevent the drastic suppression of Tri gene expression and DON biosynthesis observed in the sensitive strain.

qRT-PCR validation. To validate the RNA-seq data, nine DEGs spanning the above categories were selected for qRT-PCR analysis (Table S4). The expression trends obtained by qRT-PCR were consistent with those from RNA-seq (Figure S2), confirming the reliability of the transcriptomic dataset.

### 2.5. Metabolomic Analysis

#### 2.5.1. Global Metabolic Profiling

The global metabolic profiles of all samples were visualized via unsupervised PCA (Figure S3). The tightly clustered QC samples verified the repeatability and stability of the LC-MS detection system. The first two principal components collectively explained 56.2% of total metabolic variation. Along the PC1 axis, the control groups (R_CK, S_CK) and fungicide-treated groups (R_T, S_T) were clearly separated, demonstrating that fungicide treatment was the primary factor reshaping fungal metabolome. Along the PC2 axis, resistant and sensitive strains exhibited distinct distribution, indicating intrinsic metabolic differences between two strains regardless of treatment. Each experimental group formed an independent cluster without cross-over, confirming significantly divergent metabolic profiles among all treatments.

#### 2.5.2. Identification of Differentially Accumulated Metabolites (DEMs)

**Figure 6:**
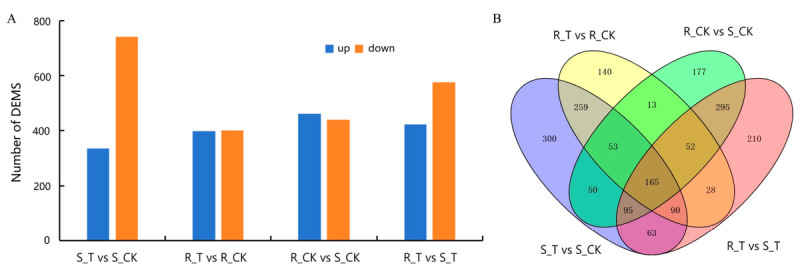
Summary of differentially accumulated metabolites (DEMs) in the four comparison groups. (**A**) Number of upregulated and downregulated DEMs in each pairwise comparison; (**B**) Venn diagram showing the overlap of DEMs across the four comparative groups.

Pairwise comparisons identified a total of 1075 DEMs in S_T vs. S_CK (740 upregulated, 335 downregulated) and 800 DEMs in R_T vs. R_CK (399 upregulated, 401 downregulated) (Figure 6A), consistent with the transcriptomic observation that the sensitive strain mounts a more extensive stress response. The comparison of the untreated controls (R_CK vs. S_CK) revealed 900 DEMs (461 upregulated, 439 downregulated), further confirming baseline metabolic divergence between the two strains. A Venn diagram showed that 165 DEMs were commonly altered across all comparisons (Figure 6B). These DEMs spanned multiple chemical classes, including carbohydrates, amino acids, lipids (fatty acids, phospholipids), terpenoids and phenylpropanoids, suggesting broad metabolic remodeling associated with resistance. The major differentially metabolites are provided in Supplementary Table S5.

#### 2.5.3. KEGG Enrichment Analysis of DEMs

**Figure 7:**
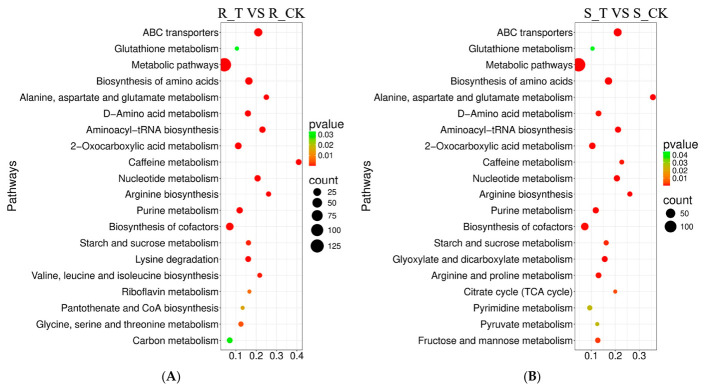

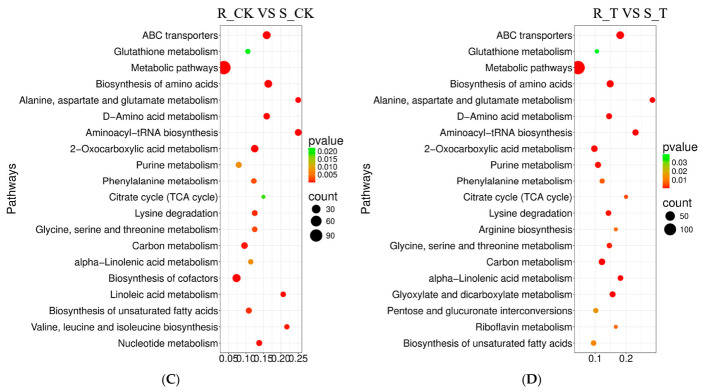
KEGG enrichment analysis of differentially accumulated metabolites in (**A**) R_T vs. R_CK, (**B**) S_T vs. S_CK, (**C**) R_CK vs. S_CK and (**D**) R_T vs. S_T. The *y*-axis represents the KEGG pathway and the *x*-axis shows the number of DEMs in each pathway. The Padj value (specified using the *p* value option) is displayed on the vertical axis, while the enrichment rate is on the horizontal axis. The size of each bubble indicates the number of enriched transcripts, which represents a KEGG pathway. Included are pathways with Padj < 0.05, which, by default, display the top 20.

The KEGG pathway enrichment of DEMs (Figure 7) revealed that the ABC transporter and glutathione metabolism pathways were universally enriched across all comparisons, mirroring the transcriptomic findings. However, comparison-specific enrichments highlighted divergent metabolic strategies. In the sensitive strain (S_T vs. S_CK), DEMs were significantly enriched in the TCA cycle, pyruvate metabolism and pyrimidine metabolism, indicative of disrupted central carbon and energy metabolism. In contrast, the resistant strain (R_T vs. R_CK) showed specific enrichment in riboflavin metabolism and pantothenate and CoA biosynthesis, pathways involved in redox homeostasis and cofactor supply. Notably, the comparison of untreated controls (R_CK vs. S_CK) revealed the enrichment in unsaturated fatty acid biosynthesis and fatty acid metabolism, suggesting that the resistant strain has a pre-existing membrane remodeling capability.

#### 2.5.4. Key Differential Metabolites Associated with Resistance

**Figure 8:**
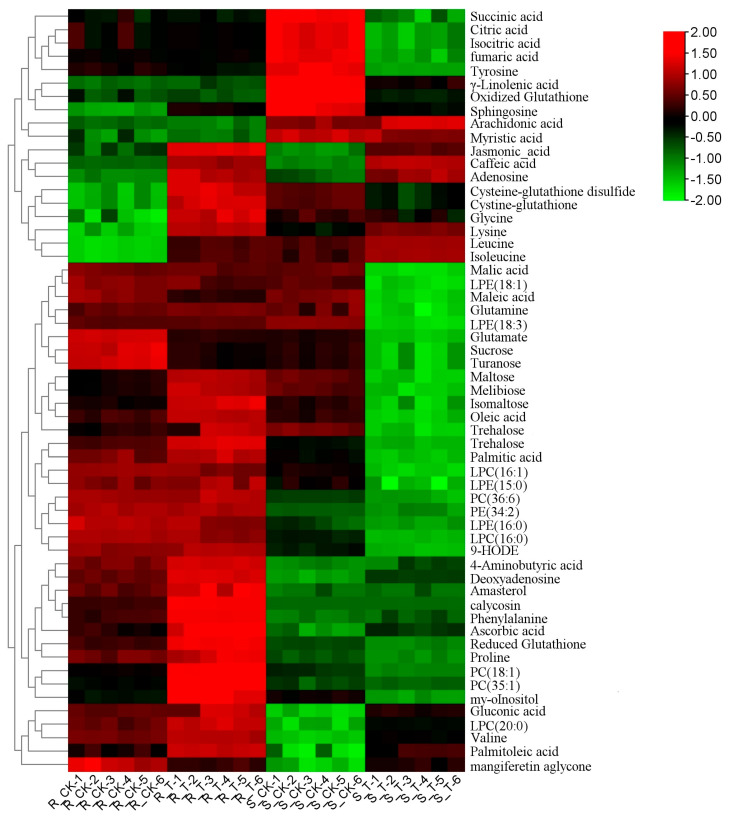
Heatmap of the key differentially accumulated metabolites implicated in resistance metabolism. Green indicates low relative abundance and red indicates high relative abundance. Colored blocks along the *y*-axis of the heatmap represent the metabolite class to which each compound belongs.

To gain a deeper insight into metabolic adaptations, we examined specific metabolites within the TCA cycle, membrane lipids and stress-related pathways (Figure 8). In the sensitive strain, pydiflumetofen treatment caused a marked accumulation of succinate alongside the depletion of citrate, malate and fumarate, confirming TCA cycle blockage at the SDH step. In contrast, the resistant strain maintained normal levels of these intermediates, indicating an uninterrupted TCA cycle flux.

Regarding membrane metabolism, the resistant strain exhibited a significant accumulation of the phospholipids phosphatidylcholine (PC) and lysophosphatidylethanolamine (LPE), as well as unsaturated fatty acids (oleic acid, palmitoleic acid) and ergosterol derivatives (amasterol) following treatment. These changes are consistent with a role in preserving membrane integrity under fungicide stress. The sensitive strain showed the opposite trend, with decreased PC and PE, consistent with its increased membrane permeability (Figure 2A). Additionally, the resistant strain accumulated high levels of the osmoprotectant proline and the antioxidant glutathione (GSH), whereas these metabolites decreased in the sensitive strain. The flavonoid calycosin was significantly accumulated in the resistant isolate upon pydiflumetofen treatment, with a 4.18-fold increase relative to its untreated control (R_CK); similarly, other phenolics (mangiferin aglycone, caffeic acid) in the resistant strain may further contribute to oxidative stress tolerance.

### 2.6. Integrative Analysis of the Transcriptome and Metabolome

To identify the core pathways orchestrating pydiflumetofen resistance, we performed an integrative analysis of the transcriptomic and metabolomic datasets. The KEGG pathway enrichment of both DEGs and DEMs consistently highlighted two pathways: ABC transporters and glutathione metabolism.

**Figure 9:**
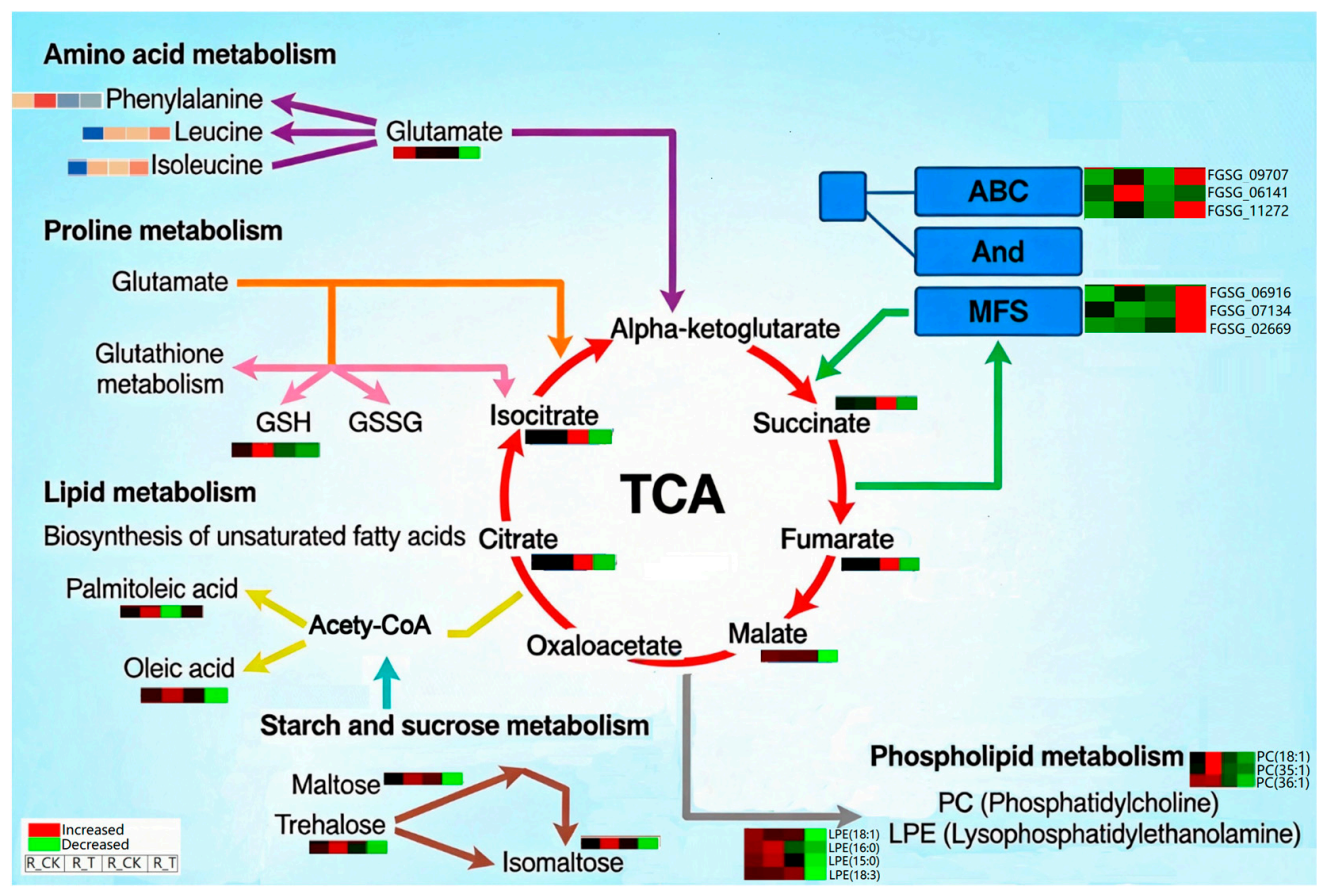
The integrated analysis of the transcriptome data and metabolome.

In the ABC transporter pathway, the multidrug efflux pump genes FGSG_00669 and FGSG_04440 were upregulated 2.3- and 1.8-fold, respectively, in the resistant isolate relative to the sensitive one under pydiflumetofen stress (Figure 9). Coinciding with this transcriptional upregulation, untargeted metabolomic profiling identified a significant accumulation of phosphatidylcholine (PC) in the pydiflumetofen-treated resistant isolate (R_T), which serves as a key lipid cofactor to enhance the efflux efficiency of ABC transporters.

In the glutathione metabolism pathway, the GST gene *FGSG_12821* showed a significantly higher induction in the resistant strain. This was mirrored by elevated levels of reduced glutathione (GSH) and its biosynthetic precursor cysteine in R_T, whereas both metabolites decreased in S_T (Figure 9). The accumulation of GSH, coupled with increased GST expression, would enhance the conjugation and detoxification of the fungicide. Furthermore, the intermediate cysteine–glutathione disulfide also trended upward in R_T, suggesting the active cycling of the glutathione pool. At the amino acid metabolism level, metabolites associated with toxin biosynthesis and stress resistance responses displayed distinct strain-specific alterations (Figure 9). Besides ABC transporter and glutathione metabolism, amino acid metabolism and other core metabolic pathways underwent dramatic alterations. The contents of aromatic amino acids (phenylalanine) and branched-chain amino acids (leucine, isoleucine) were significantly upregulated in the resistant strain, indicating that the resistant strain enhances the biosynthesis or accumulation of aromatic and branched-chain amino acids under pydiflumetofen treatment. Glutamine, a core nitrogen donor in nitrogen metabolism, was significantly decreased in the sensitive strain but remained stable in the resistant strain, suggesting that the resistant strain can better maintain nitrogen supply. Proline levels were reduced in the sensitive strain but significantly accumulated in the resistant strain. As a crucial osmoprotectant with a strong reactive oxygen species (ROS)-scavenging capacity, proline accumulation simultaneously improves the osmotic adjustment ability and oxidative damage resistance of the resistant strain, further strengthening its adaptive advantage under pydiflumetofen stress.

## 3. Discussion

This study used the field-isolated pydiflumetofen-resistant isolate W24-039 and sensitive isolate W24-016 of *F. graminearum* as experimental materials. By integrating phenotypic characterization, physiological and biochemical detection, and joint transcriptomic and metabolomic profiling, we dissected the molecular basis underlying fungal tolerance to the novel SDHI fungicide pydiflumetofen. Our results demonstrate that fixed target-site mutations in SDH subunits (FgSdhC2: C89S/A93V; FgSdhD: A21T/S30F) constitute the core genetic determinant of stable resistance in W24-039. Under fungicide stress, the resistant strain activates extensive transcriptional and metabolic remodeling, including elevated expression of detoxification and drug efflux genes and rebalanced TCA cycle and glutathione metabolism, to alleviate chemical toxicity. Together, the inherent SDH mutations and induced stress response pathways enable strong fungicide tolerance in this field strain. This multi-omics work advances our understanding of SDHI insensitivity in plant pathogenic fungi, and provides a theoretical foundation for developing rational field strategies to manage SDHI resistance.

### 3.1. Target-Site Resistance Mediated by Combined Mutations in Succinate Dehydrogenase Subunits

SDHI fungicides exert antifungal effects by blocking the ubiquinone-binding site of mitochondrial complex II. To date, point mutations in genes encoding succinate dehydrogenase (SDH) subunits have been recognized as the major resistance mechanism [18,19,20,21]. In this study, the EC_50_ value of the resistant strain W24-039 was 32 times higher than that of the sensitive strain. Sequencing detection identified compound mutations in SdhC2 (C89S, A93V) and SdhD (A21T, S30F) subunits. This mutation pattern is distinctly different from the single-point mutations reported in previous laboratory-induced resistant mutants, such as FgSdhC1 A64V and FpSdhC1 A83V/R86K [20,21,22,23]. Mutations obtained from laboratory-induced mutants are generated under short-term artificial fungicide selection, and most of these single-site substitutions only produce moderate resistance and often lead to obvious fitness defects [20,24]. Importantly, the differences in resistance level and fitness cost between lab-generated single-site mutants and our field-derived multi-mutation isolate should not be simply attributed to whether mutations originated from artificial laboratory screening or field agricultural selection. The field-resistant isolate characterized here was recovered from environments with long-term agricultural fungicide selection, and harbors multiple compound substitutions across FgSdhC2 and FgSdhD. Such multi-site combined mutations may form a functionally optimized genotype that confers high-level pydiflumetofen resistance without impairing core fungal fitness.

Notably, the four amino acid substitutions (C89S and A93V in FgSdhC2; A21T and S30F in FgSdhD) all localize to conserved transmembrane regions of the two subunits. Structural studies have established that these transmembrane helices collectively form the ubiquinone-binding pocket, the principal target of SDHI fungicides [25,26,27]. Although the precise interactions among these mutations remain unclear, their simultaneous occurrence within the same binding microenvironment is likely to induce spatial conformational rearrangements that generate additive or synergistic effects. This interpretation is supported by our physiological observations: SDH activity and intracellular ATP levels of the resistant strain remained largely stable after fungicide treatment, consistent with a structurally adapted binding pocket that maintains normal enzymatic function.

The genome of *F. graminearum* contains two paralogous genes, SdhC1 and SdhC2 [28,29]. Previous studies have shown that FgSdhC1 is expressed at an extremely low level under normal growth conditions but is significantly upregulated upon SDHI exposure. This gene is non-essential for normal growth, yet it can sustain the function of the SDH complex and confer natural resistance or reduced susceptibility to SDHIs [29]. These findings deepen our understanding of functional differentiation among SDH subunits. Transcriptomic data indicated that the sensitive strain significantly upregulated SdhC1 expression under fungicide stress, which is presumed to be a compensatory mechanism for impaired SdhC2 function [29]. However, no differentially expressed SDH subunit genes were detected in the resistant strain, suggesting that the mutated SdhC2 is sufficient to maintain the normal function of the SDH complex without additional transcriptional compensation. Furthermore, recent studies on *Fusarium asiaticum* have verified that FaSdhC1 and FaSdhC2 exhibit functional divergence in developmental regulation and pathogenicity, and genetic variations in SdhC subunits act as key determinants of fungal sensitivity and resistance to SDHIs [30,31,32]. Collectively, these results support the viewpoint that SdhC1 and SdhC2 play distinct structural or regulatory roles in mitochondrial respiratory complex II.

### 3.2. Maintenance of Cellular Homeostasis: Metabolic Basis for Resistance Without Fitness Costs

Cell membrane integrity, mitochondrial energy metabolism and deoxynivalenol (DON) biosynthesis are key physiological characteristics that determine the growth, development and pathogenicity of phytopathogenic fungi [13,33,34,35]. Our previous systematic fitness assessment of strain W24-039 revealed that this resistant strain showed no significant differences from the sensitive strain in major fitness indicators, including mycelial growth rate, sporulation quantity, spore germination rate, pathogenicity and DON production capacity [17]. The phenotype of resistance without fitness costs provides an important prerequisite for exploring its underlying metabolic regulatory mechanisms in the present study.

Physiological measurements showed that pydiflumetofen treatment triggered a sharp rise in relative electrical conductivity in the sensitive strain, indicating severe damage to cell membrane integrity, whereas the membrane function of the resistant strain remained stable. Metabolomic data provided direct molecular explanations for this phenotypic difference. After fungicide treatment, the resistant strain specifically accumulated phosphatidylcholine (PC) and phosphatidylethanolamine (PE), the major structural phospholipids of fungal cell membranes, as well as unsaturated fatty acids (oleic acid, palmitoleic acid) and ergosterol derivatives. These metabolic changes function synergistically: accumulated PC and PE maintain the barrier function of cell membranes; increased unsaturated fatty acids improve membrane fluidity under stress conditions; and ergosterol modulates membrane rigidity and permeability [36,37]. The combined effects of these metabolites greatly enhance membrane the stability and osmotic tolerance of the resistant strain under fungicide stress [33,38]. On the contrary, the contents of these protective lipids decreased significantly in the sensitive strain, which is highly consistent with its damaged cell membrane integrity.

As the direct target of SDHIs, energy metabolism represents another major difference between the two strains. SDHIs inhibit SDH activity, block the flux of the TCA cycle and disrupt the function of the mitochondrial electron transport chain [8,39]. Unlike previously reported laboratory-induced mutants, of which the SDH activity and ATP content were obviously higher than those of wild-type parental strains [20], the field-resistant strain in this study maintained SDH activity and ATP levels close to the untreated control after fungicide exposure. As the primary virulence factor of *F. graminearum*, DON biosynthesis heavily relies on acetyl-CoA and energy supplied by the TCA cycle [40,41]. Pydiflumetofen treatment caused a substantial decline in DON production in the sensitive strain. By comparison, the downregulation amplitude of Tri gene clusters (e.g., Tri4, Tri5) was much lower in the resistant strain, and DON production remained unchanged. This indicates that SDHI fungicides indirectly inhibit DON biosynthesis mainly by suppressing the energy metabolism of pathogens.

The above differences reflect the divergent selection directions between natural field selection and laboratory artificial selection. Laboratory induction tends to screen mutants with short-term fungicide tolerance, even accompanied by high fitness costs. In contrast, field natural selection preferentially retains genotypes that acquire resistance without disturbing the overall balance of metabolic networks. It has been reported that cyclobutrifluram-resistant mutants of *F. graminearum* generally suffer remarkable declines in multiple fitness traits such as mycelial growth, sporulation, spore germination, pathogenicity and mycotoxin production [20]. Nevertheless, the resistant strain in this study showed no such fitness defects in prior evaluations [17]. This phenotype is well-correlated with the stable metabolism of the resistant strain—characterized by membrane lipid protection, sustained energy homeostasis and unobstructed TCA cycle flux—indicating that these metabolic adjustments likely underpin the observed absence of fitness costs under the tested conditions.

### 3.3. Synergistic Remodeling of the Transcriptome and Metabolome: Efflux–Detoxification Axis and Reconstruction of the Core Metabolism

In addition to target-site mutations, xenobiotic detoxification and drug efflux systems play critical roles in SDHI resistance of plant pathogenic fungi [42,43,44]. Previous studies have confirmed that pydiflumetofen can induce the upregulation of xenobiotic detoxification pathways. The overexpression of ATP-binding cassette (ABC) and major facilitator superfamily (MFS) transporter genes is significantly correlated with reduced fungal susceptibility to SDHIs [45]. Transcriptomic analysis in this study showed that gene families related to detoxification and efflux, including cytochrome P450s, glutathione S-transferases (GSTs), ABC transporters and MFS transporters, were significantly enriched and expressed at much higher levels in the resistant strain than in the sensitive strain. The highly expressed ABC transporter genes (FGSG_02847, FGSG_09707, FGSG_06141, FGSG_11272, FGSG_11240, FGSG_02786) and MFS transporter genes (FGSG_03726, FGSG_01302) encode multidrug efflux pumps. These transporters utilize ATP to actively expel intracellular fungicides out of cells, thus preventing the toxic accumulation of the fungicide inside fungal cells [46,47].

Notably, metabolomic results were highly consistent with transcriptomic changes. Phosphatidylcholine, which has been proven to act as a cofactor assisting the substrate binding of ABC transporters, accumulated markedly in the resistant strain. This may accelerate fungicide efflux by improving the substrate binding efficiency of efflux pumps [48]. Meanwhile, the coordinated upregulation of GST and cytochrome P450 genes was accompanied by the significant accumulation of reduced glutathione (GSH) and its biosynthetic precursor cysteine, which, together, form an efficient binding and degradation network for detoxification [49].

Integrated multi-omics analysis further revealed prominent synergistic remodeling of core pathways, including the TCA cycle, ABC transporter-mediated efflux, glutathione metabolism and amino acid metabolism in the resistant strain. As the central hub of cellular energy and material metabolism, TCA cycle homeostasis directly governs fungal growth and development [50]. Under fungicide stress, the sensitive strain exhibited massive accumulation of succinate and decreased contents of downstream TCA intermediates (citrate, malate), which is a typical metabolic characteristic of blocked TCA flux caused by SDH inhibition. In comparison, the levels of TCA cycle intermediates in the resistant strain showed no obvious fluctuations, demonstrating that the TCA cycle flux was well maintained under stress. In contrast, transcriptomic enrichment revealed that the oxidative phosphorylation pathway was constitutively enriched in the resistant strain even under control conditions (R_CK vs. S_CK), suggesting an inherent energy metabolic advantage that may facilitate resistance without requiring acute transcriptional compensation upon fungicide challenge. Cross-validation across transcriptomics and metabolomics strongly supports our conclusion that target-site mutations protect TCA cycle function and provide sufficient energy to support the continuous growth of the resistant strain under fungicide pressure [51].

The differential reprogramming of amino acid metabolism also exerts important regulatory effects on fungicide resistance. Tyrosine, an aromatic amino acid closely associated with the secondary metabolic network of DON biosynthesis [52], was downregulated in both strains. However, the downregulation degree was far lower in the resistant strain, which is consistent with its stable DON production. Sustained glutamine levels ensured sufficient nitrogen supply for basal metabolism and mycotoxin synthesis in the resistant strain, while glutamine content decreased sharply in the sensitive strain [53]. Proline, an osmoprotectant with strong reactive oxygen species scavenging capacity, accumulated specifically in the resistant strain, which further enhanced its osmotic regulation ability and oxidative stress tolerance under fungicide treatment [54,55]. These metabolic adaptations jointly construct a multi-layered defense network, enabling the resistant strain to maintain cellular functional homeostasis under stress.

While this study provides a comprehensive molecular characterization of pydiflumetofen resistance in a representative field isolate, several avenues remain to be explored. The whole-genome sequencing of paired resistant and sensitive isolates may help uncover additional genetic factors that contribute to the resistance phenotype or its fitness compensation. Comparative metabolomic profiling with fungicides possessing unrelated modes of action will further distinguish pydiflumetofen-specific metabolic signatures from general xenobiotic defense responses. Extending such mechanistic work to community-level field investigations would ultimately clarify how resistant genotypes influence the grain mycobiota and competitive dynamics among coexisting pathogens. Addressing these questions will deepen our understanding of SDHI resistance evolution, and support the development of more sustainable management strategies.

## 4. Materials and Methods

### 4.1. Strains and Culture Conditions

A pydiflumetofen-sensitive strain (*F. graminearum* W24-016) was used as the sensitive control, and a pydiflumetofen-resistant strain (*F. graminearum* W24-039) was used as the resistant test strain. Both were isolated and identified from field samples by our laboratory during the systematic monitoring of fungicide sensitivity in *F. graminearum* populations collected from Henan Province. The two strains were first cultured on a potato dextrose agar (PDA, Qingdao Hope Bio-Technology Co., Ltd., Qingdao, China) medium at 27 °C for 5 days. Mycelial discs (8 mm in diameter) were excised from the actively growing margin of each colony, and eight discs per strain were transferred into 250 mL Erlenmeyer flasks containing 100 mL of the potato dextrose broth (PDB, Qingdao Hope Bio-Technology Co., Ltd., Qingdao, China) medium. The flasks were incubated on a rotary shaker at 170 rpm and 27 °C for 48 h, with six biological replicates prepared for each strain. Subsequently, three replicate flasks per strain were supplemented with a technical-grade pydiflumetofen (Ampuress Standard Technical Service Co., Ltd., Shanghai, China) stock solution dissolved in DMSO to a final concentration of 0.3 μg/mL, while the remaining three flasks were maintained as the untreated controls. After an additional 12 h of shaking incubation, mycelia were harvested via vacuum filtration, rinsed three times with sterile distilled water to remove residual medium, then blotted dry with sterile filter paper to remove surface moisture. Mycelia were split into two portions: one portion was immediately subjected to the determination of physiological parameters, and the other was snap-frozen in liquid nitrogen and stored at −80 °C for the subsequent transcriptome sequencing, metabolome profiling and quantitative real-time PCR (qRT-PCR) analysis of gene expression levels.

### 4.2. SDH Subunit Gene Sequencing

Genomic DNA was extracted from both resistant and sensitive strains using the CTAB method [56]. The PCR amplification of the five SDH subunit genes (FgSdhA, FgSdhB, FgSdhC1, FgSdhC2 and FgSdhD) was performed using the primer pairs designed by Chen et al. [28] (Table S6). All purified PCR products were subjected to bidirectional Sanger sequencing. Sequence alignment and amino acid variation comparison were subsequently carried out between the resistant isolate W24-039 and sensitive isolate W24-016.

### 4.3. Determination of Physiological Indicators

Fitness assessment: Fitness parameters, including mycelial growth rate, sporulation, conidial germination and pathogenicity on wheat seedlings, were compared between the resistant and sensitive isolates. All fitness evaluation protocols were consistent with our previous study [17]; pathogenicity was determined via a wheat coleoptile inoculation assay under controlled microclimate conditions.

Conductivity determination: First, 0.3 g of mycelium was weighed and transferred into a 50 mL centrifuge tube containing 20 mL of distilled water. The electrical conductivity of the suspension was measured at 20, 40, 60, 80, 100, 120, 140, 160, and 180 min post-incubation using a conductivity meter (Mettler-Toledo Instruments (Shanghai) Co., Ltd., Shanghai, China). After the 180 min measurement, the centrifuge tubes were placed in a boiling water bath for 5 min to completely disrupt cell membranes. Following cooling to room temperature, the final electrical conductivity was determined. The relative conductivity at each time point was calculated using the formula below to evaluate the effect of pydiflumetofen on the cell membrane permeability of *F. graminearum*:

$$\mathrm{Relative}\;\mathrm{electrical}\;\mathrm{conductivity}\;(\%)\;=\;\frac{Electrical\;conductivity\;of\;suspension\;at\;each\;time\;point}{Final\;electrical\;conductivity\;after\;boiling}\;\times \;100$$

Succinate dehydrogenase (SDH) and ATP activity assays: For each sample, 0.1 g of mycelium was weighed. SDH activity and ATP content were measured with the succinate dehydrogenase activity assay kit and ATP content assay kit from Solarbio Science & Technology Co., Ltd., Beijing, China. respectively, according to the manufacturer’s instructions. Each treatment was performed with three technical replicates, and the entire experiment was repeated three times independently.

Determination of DON: Freeze-dried ground mycelia (5.00 ± 0.01 g) were accurately weighed into a 50 mL polypropylene centrifuge tube and mixed with 20 mL of an acetonitrile-water-acetic acid solution (70:29:1, *v*/*v*/*v*). The mixture was vigorously vortexed for 1 min (maximum intensity) and then subjected to refrigerated centrifugation at 8000 rpm for 5 min at 4 °C. A 750 μL aliquot of the supernatant was precisely transferred into a 2 mL tube, diluted with 750 μL of high-performance liquid chromatography (HPLC)-grade water, and mixed thoroughly. After a subsequent refrigerated centrifugation at 11,000 rpm for 10 min, the solution was filtered through a 0.22 μm membrane into a sample vial. The deoxynivalenol (DON) was quantified using a liquid chromatography–mass spectrometry (LC-MS) system.

### 4.4. Transcriptome Analysis

#### 4.4.1. Transcriptome Sequencing

Total RNA was extracted using the TRIzol reagent (Invitrogen, Thermo Fisher Scientific, Carlsbad, CA, USA). The purity and concentration of the extracted RNA were determined by 1% agarose gel electrophoresis and a microvolume ultraviolet (UV) spectrophotometer. Three replicate samples were used for each treatment. RNA extraction, library construction and sequencing were performed by Biotech Biomedical Technology Co., Ltd., Shanghai, China, following standard protocols.

#### 4.4.2. Transcriptome Data Analysis and Differential Expression Gene Screening

The obtained clean reads were mapped to the *F. graminearum* reference genome (ASM24013v3, https://www.ncbi.nlm.nih.gov/genome/?term=F.+graminearum, accessed on 12 August 2025) using HISAT2 software (version 2.2.1). Pairwise transcriptome comparisons (S_T vs. S_CK, R_T vs. R_CK, R_CK vs. S_CK, R_T vs. S_T) were conducted to identify differentially expressed genes (DEGs), applying the screening thresholds of |log_2_FC| ≥ 1 and an adjusted *p*-value < 0.05. Gene Ontology (GO) functional annotation and Kyoto Encyclopedia of Genes and Genomes (KEGG, https://www.kegg.jp/, accessed on 20 August 2025) pathway enrichment analyses were performed on the DEGs to elucidate the resistance-related mechanisms.

#### 4.4.3. Validation of Candidate Genes via qRT-PCR

The relative mRNA expression levels of candidate genes were compared using quantitative real-time PCR (qRT-PCR). Total RNA was extracted using a TRIzol reagent kit. Gene sequences were obtained from the NCBI database, and primer design and specificity detection were performed using the NCBI Primer-BLAST tool (https://www.ncbi.nlm.nih.gov/tools/primer-blast/, accessed on 16 January 2026). The translation elongation factor 1α gene (*EF1α*) was used as the internal reference gene, and the nine primer sequences for the candidate genes are listed in Table S4. Complementary DNA (cDNA) was synthesized from 1.0 μg of total RNA using PrimeScript™ FAST RT reagent Kit with gDNA Eraser (Takara Biomedical Technology (Dalian) Co., Ltd., Dalian, China). qRT-PCR was performed using an ABI 7500 Real-Time PCR System (Applied Biosystems, Foster City, CA, USA). The thermal cycling program was set as follows: an initial denaturation step at 95 °C for 3 min, followed by 40 cycles of 95 °C for 30 s, 55 °C for 30 s, 72 °C for 30 s. Gene expression levels were calculated using the 2^−ΔΔCt^ method. All qRT-PCR analyses were conducted with three biological replicates and three technical replicates. Data analysis was performed using IBM SPSS Statistics 22.0 software.

### 4.5. Metabolome Analysis and Metabolite Extraction

#### 4.5.1. Metabolite Extraction

Lyophilized fungal mycelial samples (25 ± 1 mg) were mixed with grinding beads and 500 μL of an extraction solvent (MeOH:ACN:H_2_O, 2:2:1, *v*/*v*/*v*) containing deuterated internal standards. The mixtures were vortexed for 30 s, homogenized (35 Hz, 4 min), and sonicated for 5 min in a 4 °C water bath. This homogenization–sonication cycle was repeated three times. The samples were then incubated at −40 °C for 1 h to precipitate proteins, followed by centrifugation at 12,000 rpm for 15 min at 4 °C. A 120 μL aliquot of the supernatant was transferred into a 2 mL LC-MS vial. The quality control (QC) samples were prepared by pooling 10 μL aliquots from each sample to monitor instrument stability. Six biological replicates were processed for each treatment.

#### 4.5.2. LC-MS/MS Analysis

Untargeted metabolomic profiling was performed using a Vanquish UHPLC system coupled to an Orbitrap Exploris 120 mass spectrometer (Thermo Fisher Scientific, Waltham, MA, USA). Chromatographic separation was achieved on a Phenomenex Kinetex C18 column (Phenomenex, Torrance, CA, USA) (2.1 mm × 100 mm, 2.6 μm) maintained at 25 °C. The mobile phase consisted of 0.01% acetic acid in water (A) and isopropanol:acetonitrile (1:1, *v*/*v*) (B). The autosampler temperature was set to 4 °C, and the injection volume was 2 μL. The mass spectrometer operated in information-dependent acquisition (IDA) mode via Xcalibur software (version 4.7, Thermo Fisher Scientific, Waltham, MA, USA). Electrospray ionization (ESI) source parameters were set as follows: sheath gas flow rate, 50 Arb; aux gas flow rate, 15 Arb; sweep gas, 1 Arb; capillary temperature, 320 °C; and vaporizer temperature, 350 °C. The spray voltage was 3.8 kV in the positive mode and −3.4 kV in the negative mode. Full MS resolution was set to 60,000, and MS/MS resolution to 15,000, with the stepped normalized collision energies (SNCE) of 20, 30 and 40.

#### 4.5.3. Metabolite Quantitative Analysis

Raw MS data were converted to mzXML format using ProteoWizard (version 3.0.22281), and peak detection with an in-house R script implemented using the XCMS package, extraction, alignment and integration. Metabolite annotation was performed utilizing the BiotreeDB (V3.0) database. Data were normalized using the total ion current method. The resulting dataset was imported into SIMCA 18.0.1 (Sartorius Stedim Data Analytics AB, Umeå, Sweden) for multivariate statistical analysis. Following scaling and logarithmic transformation, Principal Component Analysis (PCA) was conducted to visualize sample distribution. Orthogonal Partial Least Squares Discriminant Analysis (OPLS-DA) was utilized to calculate the Variable Importance in Projection (VIP) scores. Metabolites with a VIP > 1 and *p* < 0.05 (Student’s *t*-test) were defined as significantly differential metabolites. Pathway enrichment analysis was subsequently performed using the KEGG and MetaboAnalyst databases (version 6.0).

## 5. Conclusions

This study provides a systems-level characterization of the molecular mechanisms underlying field-evolved pydiflumetofen resistance in *F. graminearum*, with three key novel findings: (1) a novel set of compound mutations in FgSdhC2 (C89S/A93V) and FgSdhD (A21T/S30F) was identified, which preserve SDH enzymatic activity and mitochondrial energy homeostasis under pydiflumetofen stress without fitness costs; (2) resistance is not a single target-site event but a multi-faceted mechanism mediated by the synergistic interaction between target mutations and a genome-wide “efflux–detoxification axis” consisting of ABC/MFS transporters and P450/GST detoxification enzymes; and (3) ABC transporter and glutathione metabolism pathways were pinpointed as the core synergistic hubs coordinating resistance, with phosphatidylcholine and reduced glutathione serving as key metabolic cofactors to enhance efflux and detoxification efficiency. These findings break the traditional linear “mutation resistance” paradigm and provide a theoretical framework for the development of sustainable SDHI resistance management strategies, such as the identification of key efflux–detoxification components as potential targets for synergists that could be explored in future combination studies, in addition to establishing molecular detection markers for the novel SDH compound mutations in field *F. graminearum* populations.

## Figures and Tables

**Table 1:** The sensitivity of *Fusarium graminearum* to pydiflumetofen.

Strain	Phenotype	EC_50_ (μg/mL)	Mutation Type
W24-039	R	2.460 ± 0.16 b	SdhC_2_(C89S/A93V), SdhD(A21T/S30F)
W24-016	S	0.077 ± 0.03 a	-

Note: R = pydiflumetofen-resistant strain; S = pydiflumetofen-sensitive strain. EC_50_ values are expressed as the mean ± standard deviation of three biological replicates. Lowercase letters a and b denote statistically significant differences among strains in the same column at the 0.05 probability level. The symbol “-” indicates that no amino acid substitution mutations were identified in the SdhC and SdhD genes.

**Table 2:** The biological characteristics of *F. graminearum* strains with different sensitivities to pydiflumetofen.

Strain	Phenotype	Colony Diameter/cm	Sporulation/(10^5^ Spores/mL)	Lesion Size/cm
W24-016	S	7.59 ± 0.12 a	9.87 ± 0.25 a	1.06 ± 0.11 a
W24-039	R	7.23 ± 0.14 a	9.89 ± 0.28 a	1.09 ± 0.15 a

Different letters in the same column indicate significant difference at *p* < 0.05 by LSD test.

## Data Availability

The original contributions presented in this study are included in the article/Supplementary Materials. Further inquiries can be directed to the corresponding authors.

## Supplementary Materials

The following supporting information can be downloaded at: https://www.mdpi.com/article/10.3390/ijms27156685/s1.
