# Supplementary material for: Transcriptome and Metabolome Dissection of Multilayered Pydiflumetofen Resistance Mechanisms in Fusarium graminearum
Source: Int J Mol Sci. 2026 Jul 27;27(15):6685. doi: 10.3390/ijms27156685 (PMC13466391; doi:10.3390/ijms27156685)
Supplement: Supplementary file 1 [file ijms-27-06685-s001.zip › ijms-4396822-supplementary.pdf]

**Table S1.** Output data of RNA-seq reads in four libraries by RNA sequencing

| Sample | Raw reads | Raw bases(G) | Clean reads | Clean bases(G) | Error rate(%) | Q20(%) | Q30(%) | GC content(%) |
|---|---|---|---|---|---|---|---|---|
| S_CK_1 | 56885572 | 8.59 | 56615230 | 8.526 | 0.01 | 99.42 | 96.98 | 52.35 |
| S_CK_2 | 53712614 | 8.11 | 53489370 | 8.063 | 0.01 | 99.47 | 97.14 | 52.19 |
| S_CK_3 | 52391628 | 7.91 | 52144846 | 7.856 | 0.01 | 99.41 | 96.92 | 51.94 |
| R_CK_1 | 49706766 | 7.51 | 49434754 | 7.458 | 0.01 | 99.4 | 96.98 | 53.31 |
| R_CK_2 | 53769050 | 8.12 | 53488296 | 8.068 | 0.01 | 99.43 | 97.09 | 53.34 |
| R_CK_3 | 39224338 | 7.92 | 49050570 | 7.889 | 0.01 | 99.4 | 96.83 | 52.94 |
| S_T_1 | 49304994 | 7.45 | 49086870 | 7.403 | 0.01 | 99.45 | 97.08 | 52.23 |
| S_T_2 | 54334654 | 8.2 | 54077274 | 8.156 | 0.01 | 99.44 | 97.19 | 52.21 |
| S_T_3 | 46919698 | 8.57 | 46733276 | 7.939 | 0.01 | 99.37 | 96.72 | 52.55 |
| R_T_1 | 66305838 | 10.01 | 66090858 | 9.968 | 0.01 | 99.46 | 97.14 | 52.41 |
| R_T_2 | 56557272 | 8.54 | 56386308 | 8.503 | 0.01 | 99.49 | 97.29 | 52.4 |
| R_T_3 | 64034850 | 9.8 | 63835646 | 8.754 | 0.01 | 99.43 | 97.03 | 52.1 |

**Table S2.** Summary of sequencing reads mapping to the reference genome and quality parameters

| Sample | Clean Reads | Total Mapped | Multiple Mapped | Uniquely Mapped | Map to sense strand | Map to antisense strand |
|---|---|---|---|---|---|---|
| S_CK_1 | 56615230 | 55018844 (97.18%) | 200353 (0.36%) | 54818491 (99.64%) | 27498592 (49.98%) | 27520252 (50.02%) |
| S_CK_2 | 53489370 | 52034325 (97.28%) | 180821 (0.35%) | 51853504 (99.65%) | 26011392 (49.99%) | 26022933 (50.01%) |
| S_CK_3 | 52144846 | 50680836 (97.19%) | 167112 (0.33%) | 50513724 (99.67%) | 25334187 (49.99%) | 25346649 (50.01%) |
| R_CK_1 | 49434754 | 48190056 (97.48%) | 193322 (0.40%) | 47996734 (99.60%) | 24083422 (49.98%) | 24106634 (50.02%) |
| R_CK_2 | 53488296 | 52199951 (97.59%) | 196881 (0.38%) | 52003070 (99.62%) | 26085877 (49.97%) | 26114074 (50.03%) |
| R_CK_3 | 49050570 | 48121269 (97.62%) | 128885 (0.34%) | 37992384 (99.66%) | 19052487 (49.98%) | 19068782 (50.02%) |
| S_T_1 | 49086870 | 41647288 (84.84%) | 125914 (0.30%) | 41521374 (99.70%) | 20808715 (49.96%) | 20838573 (50.04%) |
| S_T_2 | 54077274 | 45795568 (84.69%) | 136482 (0.30%) | 45659086 (99.70%) | 22883451 (49.97%) | 22912117 (50.03%) |
| S_T_3 | 46733276 | 41252183 (85.08%) | 125628 (0.31%) | 31156555 (99.69%) | 15611978 (49.95%) | 15640205 (50.05%) |
| R_T_1 | 66090858 | 56236158 (85.09%) | 315631 (0.56%) | 55920527 (99.44%) | 28081244 (49.93%) | 28154914 (50.07%) |
| R_T_2 | 56386308 | 47971615 (85.08%) | 262971 (0.55%) | 47708644 (99.45%) | 23959491 (49.95%) | 24012124 (50.05%) |
| R_T_3 | 63835646 | 52632940 (86.17%) | 121293 (0.31%) | 38511647 (99.69%) | 19279350 (49.90%) | 19353590 (50.10%) |

**Table S4.** Primers used for analysis of gene expression by Real Time -qPCR.

| Primer name | Sequence 5′ -3′ |
|---|---|
| FgsdhC1-F(FGSG_01981) | TAGCAACTTCATCGCGTCCA |
| FgsdhC1-R(FGSG_01981) | TCGAGGTTTGTGATGGTGGG |
| FgsdhC2-F(FGSG_09012) | GTTCCGTCAAGGTCTCCCAA |
| FgsdhC2-R(FGSG_09012) | GGCGATGGAGAAGCCGTAGA |
| FgsdhD-F(FGSG_00743) | CTGCTTTCCACAACACGACC |
| FgsdhD-R(FGSG_00743) | TAGGGTTGAGAGAGCCAGCA |
| FgTri5-F(FGSG_03537) | GTCCGAGAGCTACGACAAGG |
| FgTri5-R(FGSG_03537) | AGTTCCATAGACCAGCCCCT |
| Cytochrome c oxidase-F (FGSG_01739) | CTACCAGAACGCTTACAAGA |
| Cytochrome c oxidase-R (FGSG_01739) | GCTGCGAGGGCCAATCTTCC |
| Glutathione S-transferase-F(FGSG_13223) | GCTGCCCCCGTCATCCAAGA |
| Glutathione S-transferase-R(FGSG_13223) | GCTGCCCCCGTCATCCAAGA |
| ABC transporter-F(FGSG_09707) | CGTTTGCATGTATCGCCCTG |
| ABC transporter-R(FGSG_09707) | GCCATCATCGGATTCGTCCT |
| MFS-type efflux pump-F(FGSG_06336) | GTTTGCTCAGTGGAGTCGGA |
| MFS-type efflux pump-R(FGSG_06336) | AGATCAAGCACAAGACGCCA |
| Cytochrome P450 monooxygenase-F(FGSG_01739) | AACGAGGTTCTGCGGCTTTA |
| Cytochrome P450 monooxygenase-R(FGSG_01739) | GTTCGAAATATGCGCTGCGT |

**Table S6.** Primer information for SDH gene amplification

| Primer name | Sequence 5′ -3′ |
|---|---|
| FgsdhA-F | ATGATGGCTTCTTCTATGG |
| FgsdhA-R | TTAGTATGTACGCTTGAAAG |
| FgsdhB-F | ATGGCTGCTCTCCGATCTTCCTC |
| FgsdhB-R | CTAGTTACCGAAAGCCATC |
| FgsdhC1-F | ATGTCACTGATCAATGTATT |
| FgsdhC1-R | CTACAACCAGAATGCCAATAC |
| FgsdhC2-F | ATGCTCGCTCAACGTGTTGG |
| FgsdhC2-R | TTACAGGAAAGCAACCAGACC |
| FgsdhD-F | ATGGCCTCAATTGCGCGTTC |
| FgsdhD-R | TTACGCCTTCCAGACTCGTC |

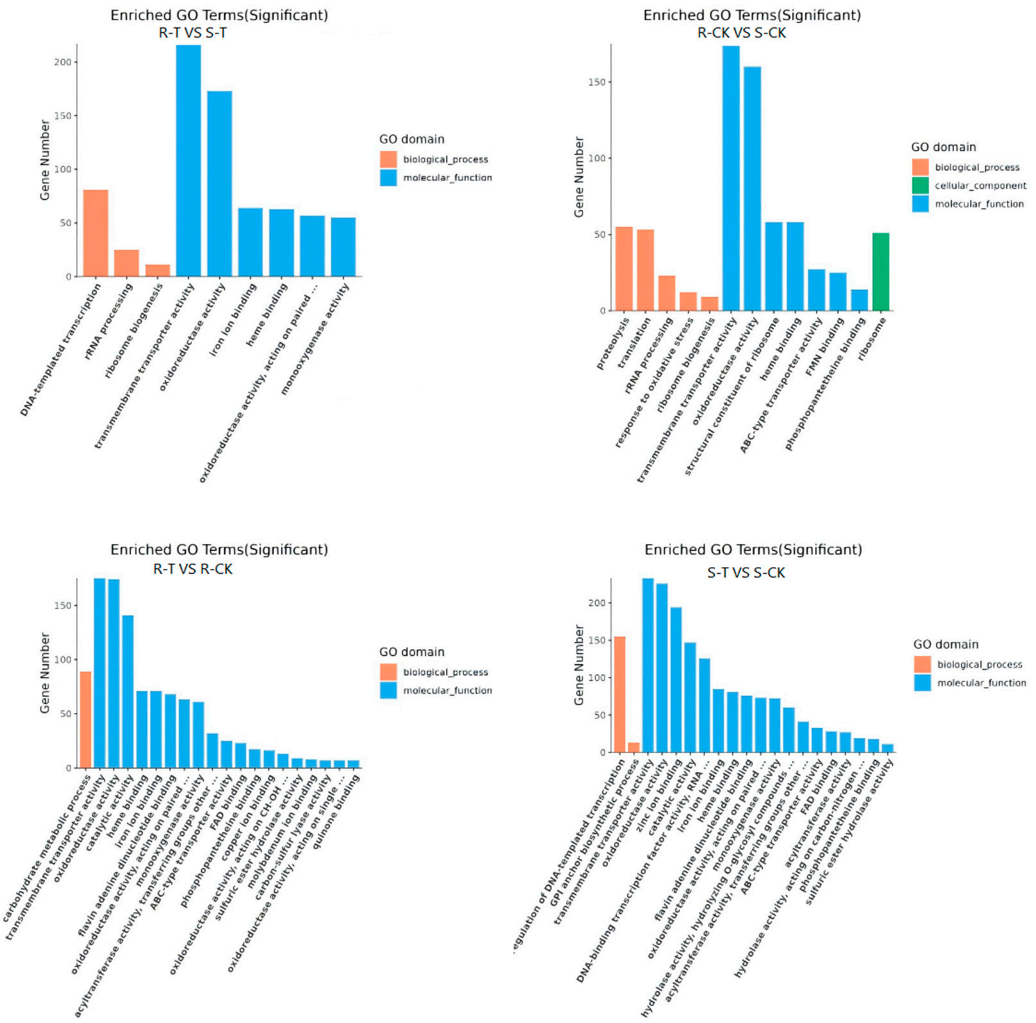


**Figure S1.** GO enrichment histogram. Note: horizontal is number of DEGs， ordinate is GO term ．Different colors are used to distinguish Biological Process(BP), Cellular Component(CC) and Molecular Function(MF).

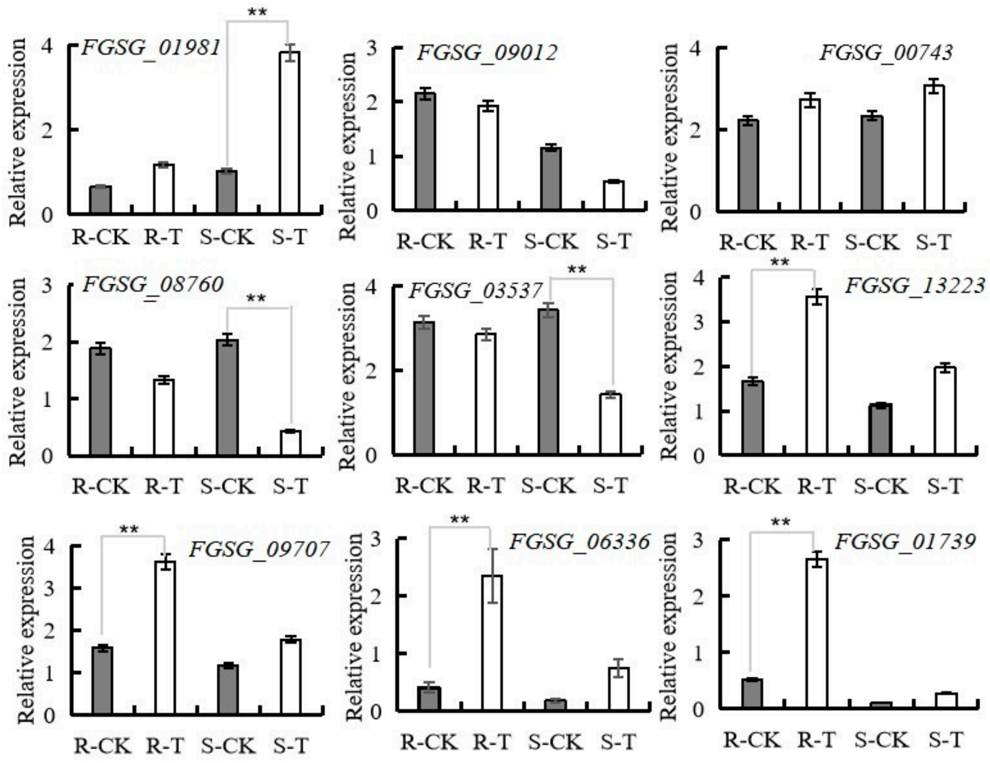


Figure S2. The relative expression levels of resistance related genes in different resistant strains. The reactions were performed in triplicate.

Note: Each data represents the means ±SD of three replicates.

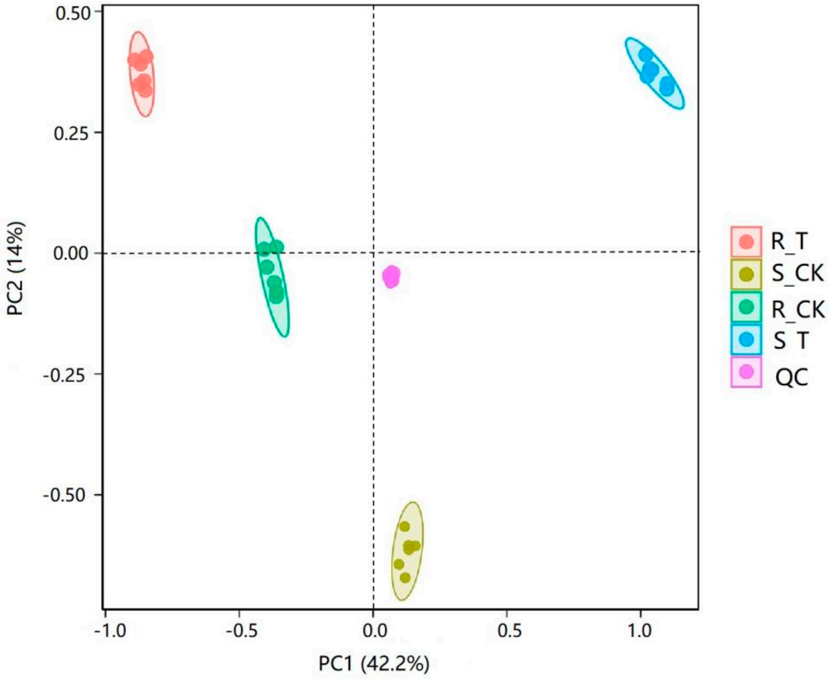


Figure S3. Principal component analysis (PCA) score plot of metabolomic profiles from resistant and sensitive strains under control and fungicide treatment.

Note: PC1 and PC2 explained 39.1% and 14.7% of the total metabolic variance, respectively. R_CK: resistant strain without fungicide treatment; S_CK: sensitive strain without fungicide treatment; R_T: resistant strain treated with fungicide; S_T: sensitive strain treated with fungicide; QC: quality control pooled samples. Independent 95% confidence ellipses were drawn for each experimental group. Tight clustering of QC samples near the origin indicated stable instrumental detection. Obvious separation between four groups revealed distinct metabolic profiles induced by strain genotype and fungicide exposure.
